# Role of Internal and External Museum Environment in Increasing Visitors’ Cognitive/Affective/Healthy Experiences and Loyalty

**DOI:** 10.3390/ijerph16224537

**Published:** 2019-11-16

**Authors:** Heesup Han, Soyeun Lee, Sunghyup Sean Hyun

**Affiliations:** 1 College of Hospitality and Tourism Management, Sejong University, 98 Gunja-Dong, Gwanjin-Gu, Seoul 143-747, Korea; heesup.han@gmail.com (H.H.); lsy2you82@hotmail.com (S.L.); 2 School of Tourism, Hanyang University, 17 Haengdang-dong, Seongdong-gu, Seoul 133-791, Korea

**Keywords:** internal museum environment, external museum environment, loyalty, involvement, knowledge value, satisfaction, desire

## Abstract

There has not been much research on the impact of the museum environment on the formation of visitor loyalty. The purpose of this study is to discover the convoluted relationships among internal and external physical environments, involvement, knowledge value, satisfaction, and desire in forming visitor loyalty. A field survey was carried out at museums. A confirmatory factor analysis with the collected data showed that the measures used included an adequate level of measurement quality. The proposed model was revised by adding four meaningful approaches to improve the anticipatory ability and model fit. Results from the structural analysis demonstrated the criticality of both internal and external dimensions of physical environments in loyalty formation and identified the significant mediating impact of cognitive, evaluative, and motivational factors in our theoretical framework. Moreover, the relative importance of desire in increasing loyalty was found. Research contributions to the museum literature are discussed.

## 1. Introduction

The function of museums in a classical fashion is providing cultural heritage to visitors [1]. Yet, it is critical to notice that museums recently face fierce competition with other educational/leisure institutions (e.g., theatres, theme/leisure parks, other rival museums, cinemas) [2]. It is also important to note that financial support from governments and other external infusion/outer sources are decreasing [1,3]. Given this competitive market environment, today, museums place great efforts on gaining popularity and attracting visitors to gain customers and revenues [2,3,4].

There has been a significant change in the paradigm of museums [1,4]. In recent decades, museums have evolved from classical assets-centered (e.g., conservation, research, exhibition) to visitor-centered (e.g., enjoyment, well-being recreation, mental health, knowledge gaining, social gaining/interaction, pleasurable experiences) [1,5,6] and from product-centered to service-centered [4,6]. Based on this shift of paradigm, museum operators strive to center on creating value and to offer satisfactory and healthy experiences to their patrons [7]. In addition, by understanding the criticality of indoor/outdoor environment quality, museum practitioners are eager to place diverse resources on the creation of visitor-friendly and healthy physical environments [8], which eventually contributes to visitors’ loyalty generation. Under the competitive museum marketplace, comprehending visitors’ loyalty generation process and uncovering the triggers of loyalty can be undeniably a crucial facet of successful museum management.

In the existing literature, a few gaps exist, which elicits the need of this research. First, although visitor loyalty at museums is a timely topic under the competitive market environment of the museum industry, little research has examined museum visitors’ post-purchase behaviors. Second, while a museum’s physical environment or atmospherics is emerging as a vital topic [9], its role relationship with other cognitive factors and satisfaction has been hardly uncovered. Third, the employment of involvement, knowledge value, and satisfaction to the museum ambience suggests interesting and important insights for both industry and academia [1,10,11,12], but scant research has been performed on the facilitating empirical influence of these essential variables in museums on visitors’ loyalty formation. Fourth, while the motivational process, as a personal factor, is a critical facet of museum experiences [5], previous research has overlooked the role of desire in visitor loyalty generation process. Fifth, to the best of our knowledge, no research has investigated the combined roles of physical environments, cognitive factors, as well as the evaluative and motivational processes in generating visitor loyalty in the museum industry. 

Overall, an understanding of museum visitors’ loyalty generation processes is somewhat narrow. The present study intends to fill these existing research gaps in a museum context. Particularly, in an empirical manner, we attempted (1) to develop a robust loyalty model of comprising physical environments, involvement, knowledge value, visitor satisfaction, and desire in museums, (2) to identify the role of internal and external physical environments in building up loyalty, (3) to explore the potential mediating effect of cognitive, evaluative, and motivational factors in the proposed theoretical framework, and (4) to compare the relative effectiveness of study variables in determining visitor loyalty.

## 2. Conceptual Framework 

### 2.1. Internal and External Physical Environment at Museums

Physical environments are important in museums as they are unique places where customers interact/communicate with diverse exhibits (e.g., heritage, art, scientific, and historical) in a limited architectural space [13]. Under the severe competition of the tourism/leisure marketplace, museum practitioners in recent years vigorously make endeavors in generating comfortable/healthy internal and external physical environments for their customers [9]. The internal environment refers to the indoor physical surroundings that influence individuals inside of a building. Both visitors and workers are under the influence of the internal physical environment. In the museum context, Shuang et al. [8] and Jeong and Lee [13] indicated that the principal elements of the indoor museum environment include thermal quality (e.g., temperature and humidity), air quality, lighting, and aesthetic environment. This internal physical environment and its performances have been recently regarded as an important aspect of services that visitors experience in museums [8,14]. 

The external environment that refers to outdoor atmospherics is also crucial as it influences visitor experiences [15,16]. In museums, such external physical environment factors as architectural style, positioning of entrances, and exterior décor and signage can be a crucial facet of external atmospherics affecting visitor experiences [9,16]. The external environment (e.g., spacious design, pretty landscape design, outdoor natural surroundings) is a constituent of the tangibility aspect of museum performances [17]. These external environment factors and internal factors together create perceived physical environments that visitors and staff cognitively/emotionally/physiologically respond to [8,13]. 

### 2.2. Involvement, Knowledge Value, Satisfaction, and Desire

Involvement refers to one’s perceived level of application, absorption, and attention stimulated by a particular behavior/experience [18]. An individual’s high involvement state implies that he/she is entirely absorbed/concentrated on the behavior/experience [12,18,19]. Regarding knowledge value, one of the main benefits/advantages that customers get at museums is acquiring particular knowledge about art, culture, or heritage [1,4]. Siu et al. [4] described such benefits/advantages related to the knowledge gained by visiting a museum as knowledge value. This value is particularly important in museums in that creating knowledge is an essential process to build visitors’ level of commitment to the museum [4] and learning about new things is an important dimension of pull motivation that drives satisfaction and loyalty to the museum [20]. In terms of satisfaction, it is essential to every company’s success, and it accordingly has long been considered as a fundamental concept in consumer behavior [21,22,23]. Dirsehan [1] conceptualized satisfactions as patrons’ evaluation of their goods/service experiences. If the experiences are better than patrons’ expectations, their satisfaction level becomes high [24]. Lastly, desire is another key concept in customer post-purchase behaviors [25]. According to Perugini and Bagozzi [26], desire indicates one’s frame of mind in which he/she has an inspiration to engage in a certain action with a goal.

### 2.3. Visitor Loyalty

While the definition of customer loyalty varies, researchers have evolved two main approaches, namely behavioral and attitudinal approaches [27,28,29,30]. According to the behavioral aspect, loyal customers are those who simply purchase a product/service repeatedly [30]. This behavioral view, relying solely on repeat patronage, ignores individuals’ intricate decision-making/evaluation process for a product/service [30,31]. That is, one’s repeat patronage does not always result from a positive attitude and commitment for a product/service. On the contrary, the attitudinal approach describes faithfulness patrons as those who make a repeat purchase with psychological dedication to the product/service [31]. Individuals’ intention/inclination to acquire a product/service again in the future and their decision to recommend it and engage in positive word-of-mouth activities are major constituents of loyalty with an attitudinal view [30,32,33]. This attitudinal approach is more commonly employed in marketing and consumer behavior [24,34]. Given this, the present research focused on the attitudinal approach when conceptualizing and measuring visitor loyalty.

### 2.4. Impact of Physical Environments on its Outcome Variables

To date, the need to understand how the physical environment affects visitors’ and workers’ behaviors has been increasingly stressed in the museum industry [8,13]. Within the marketing context of cultural heritage, performances of physical environments are considered to be key in inducing favorable cognitive evaluation and driving visitor satisfaction [1,5,20]. Particularly, Falk and Dierking [5] indicated that the important museum experience includes the personal factor comprising motivation and knowledge; and the interplay among this factor with physical factors (e.g., architecture, layout, facilities, and other activities) and social factors (e.g., social connections within visitor groups or connections between visitors and museum staff) creates visitors’ experiences. Jeong and Lee [13] investigated the museum physical surrounding and its effect on visitors’ overall satisfaction. Their findings revealed that exhibition and ambient environments and museum size exerted a significant influence on perceived learning, aesthetic experience, enjoyment, and perceived fatigue, and these relationships together increase visitors’ general gratification with museum experiences. 

In their consideration of museum visitor experiences and post-purchase behavior, Dirsehan [1] found that visitor experience influences cognitive evaluation (e.g., education in museums) and satisfaction evaluation in the process of generating revisit intent, word-of-mouth commendation, and visit increase; sensory experience was one of the key aspects of such visitor experiences in museums. In their research, the sensory dimension of visitor experiences comprised visually interesting elements and museum environments that appeals to visitors’ senses (e.g., sight, hearing, touch, and smell). Robust findings in marketing and consumer behavior also showed that diverse atmospheric cues in a service consumption situation provide various sensory stimuli to customers, helping them evaluate their consumption experiences cognitively and affectively [35,36,37]. Overall, these studies discussed above indicate that physical environments and cognitive factors are highly related, and visitors’ satisfaction is probable to be affected by such physical surroundings performance and cognitive processes. Accordingly, we predict the following:

**Hypothesis 1** **(H1).** Internal museum environment positively and significantly affects involvement.

**Hypothesis 2** **(H2).** Internal museum environment positively and significantly affects knowledge value.

**Hypothesis 3** **(H3).** External museum environment positively and significantly affects knowledge value.

**Hypothesis 4** **(H4).** Internal museum environment positively and significantly affects visitor satisfaction.

**Hypothesis 5** **(H5).** External museum environment positively and significantly affects visitor satisfaction.

### 2.5. Relationships among Involvement, Knowledge Value, and Satisfaction

Within the museum and cultural tourism contexts, individuals’ perceived level of value [11] and involvement [12] has emerged as an important concept for successful marketing and management. Chen [38] asserted that individuals’ involvement/flow evokes a positive effect. Skadberg and Kimmel [39] indicated that one’s involvement/flow experience influences augmented knowledge and his/her attitude and behavior. Siu et al. [4] attempted to explore the influence of relationship investment on commitment through knowledge and relational value. Their findings indicated that the creation of knowledge value is a vital procedure in the formation of museum visitors’ level of commitment. In their research, this commitment was evaluated with revisit and loyalty intention. In investigating visitors’ museum experiences and their likelihood to return to the museum, Brida et al. [20] also empirically demonstrated that learning about new things as a pull motivation factor is critical in generating satisfaction in the loyalty formation. Greater understanding of involvement-satisfaction and knowledge value-satisfaction relationships can inform the predictive ability of visitors’ post-purchase behavior. As a result, we proposed the following:

**Hypothesis 6** **(H6).** Involvement positively and significantly affects visitor satisfaction.

**Hypothesis 7** **(H7).** Knowledge value positively and significantly affects visitor satisfaction.

### 2.6. Relationships among Satisfaction, Desire, and Loyalty

Numerous studies in various sectors have shown that satisfaction and desire are of importance in generating one’s loyalty. In his research about exploring the role of satisfaction, Oliver [34] demonstrated that satisfaction is a crucial element of customer loyalty. Consistent with Han and Yoon [25], satisfaction exerted a significant influence on desire toward a certain action. Their finding also revealed that this desire significantly affects customer loyalty in the hotel sector. In addition, Perugini and Bagozzi [26] uncovered that desire is a direct dominance of one’s intentions for a certain consumption behavior. Findings of these studies also indicated that satisfaction and desire act as significant mediators within the decision-making structure with self-interest motives. Attributable to the significant mediating role of satisfaction and desire, the direct relationships between their antecedents (e.g., cognitive and affective factors) and its outcome variables (intention/conation/behavior) were scarcely hypothesized in prior studies [25,40,41]. Given these, it can be posited that visitor satisfaction is considerably related to desire, and effectively dealing with this satisfaction–desire relationship contributes to the increase in visitor loyalty in museums.

**Hypothesis 8** **(H8).** Visitor satisfaction positively and significantly affects desire.

**Hypothesis 9** **(H9).** Visitor satisfaction positively and significantly affects visitor loyalty.

## 3. Methods

### 3.1. Measurement Instruments and Survey Questionnaire Development 

In this research, we employed the measures previously validated in the extant literature [4,9,24,35,42,43,44,45,46,47,48,49] and amended them to be suitable in a museum context in order to evaluate constructs in the proposed conceptual model. Multiple measurement items and a seven-point scale were adopted for the evaluation of all study variables. Particularly, we utilized eight items and four items to assess internal and external museum environments, respectively. These items were adopted from Bitner [42], Forrest [9], and Han [35]. A total of three items were applied from Webster et al. [49] and Koufaris [44] to measure involvement. To evaluate knowledge value, we utilized four measurement items adopted from Nambisan and Baron [46] and Siu et al. [4]. Visitor satisfaction was measured with three items used from Han [35] and Oliver and Swan [47]. Three items were used to evaluate desire and they were adopted from Perugini and Bagozzi [48]. Lastly, visitor loyalty was assessed with five items applied from Oliver [24] and Perugini and Bagozzi [48]. The initial version of the questionnaire included these measures after modification along with a study description and questions for personal characteristics. We conducted a pre-test with academics in hospitality and tourism to increase content validity. An amendment was made based on their feedback. After this process, experts in the museum industry thoroughly reviewed the revised questionnaire and completed the final version of the survey questionnaire along with some small changes. 

### 3.2. Data Collection Process

We conducted a visitor survey at seven major museums located in the Seoul metropolitan area. Students who received survey training distributed the questionnaires to visitors in the rest areas of these museums. A detailed explanation about the study objectives was given to those who wished to participate in the survey. The sample population includes general museum visitors in Korea. Only those visitors who visit major museums in Korea at least once a year on average were eligible to participate in this survey. All participants were advised that their responses were kept both confidential and anonymous. We requested the respondents to fill out the survey questionnaire and return it onsite for a better response rate. In addition, well trained students checked the completeness of the questionnaire for the enhancement of high usable rate. A total of 310 complete questionnaires were obtained. Among them, we excluded five problematic multivariate outliers (Mahalanobis *D*^2^ (30) > 59.703, *p* < 0.001). After the removal of such extreme cases, our final data set included 305 respondents.

### 3.3. Demographic Profile of the Samples

Among the 305 survey participants, about 60.6% were female respondents, and 38.4% were male respondents. The survey participants were asked to indicate their annual incomes. Most of the respondents reported income of 25,000,000 to 69,999,999 Korean won (46.8%), followed by 24,999,999 won or less (30.0%), and 70,000,000 won or more (23.2%). When their education level was queried, about 70.6% of the respondents have a bachelor’s degree, followed by graduate-degree holders (14.2%), 2-year college or some college graduates (8.1%), and high-school graduates or less (6.1%). The survey asked participants to report the frequency of museum visit. The largest group visits a museum about 2–5 times a year (51.0%), about 32.3% reported that their visit frequency is once a year or less, approximately 11.0% indicated 6–10 times a year, and about 5.7% reported that they visit a museum 11 times a year or more. Lastly, survey participants’ mean age was 31.6 years. The age of the respondents was between 19 and 72 years old.

### 3.4. Data Analysis

In the present research, we utilized IBM SPSS Statistics 20 and AMOS 20 (Armonk, NY, USA) for Windows for all statistical analyses. Descriptive statistics were utilized to identify the sample characteristics. To achieve research objectives, we followed Anderson and Gerbing’s [50] two-step procedure involving the measurement and structural model assessments. Specifically, we tested by employing a confirmatory factory analysis (CFA) with the maximum likelihood estimation approach for data quality comprising composite reliability and construct validity. The proposed conceptual framework was then estimated using a structural equation modeling (SEM) along with the maximum likelihood estimation method. Subsequently, the aforementioned hypotheses were tested on the basis of the results from the SEM.

## 4. Results and Hypotheses Testing

### 4.1. Data Quality Assessment

We generated the measurement model using the CFA. Our result from the CFA indicated that the measurement model included an adequate fit to the data (χ^2^ = 1092.539, *df* = 380, *p* < 0.001, χ^2^/*df* = 2.875, RMSEA = 0.079, CFI = 0.915, IFI = 0.915, TLI = 0.902). Each latent variable’s loadings were significant at a 0.01 level. Internal consistency for each research construct was assessed. As shown in Table 1, results of the composite-reliability testing revealed that all reliability values were greater than Bagozzi and Yi’s [51] suggested cut-off of 0.600 (internal museum environment = 0.858; external museum environment = 0.841; involvement = 0.924; knowledge value = 0.943; visitor satisfaction = 0.931; desire = 0.954; and visitor loyalty = 0.920). The result proved the internal consistency of the measures for all variables used in the present study. Consequently, construct validity was examined. Our calculation of the average variance extracted (AVE) generally supported the convergent validity as AVE of research constructs were close to or above the recommended value of 0.500 [52]. As shown in Table 2, discriminant validity was also supported such that the AVE values were greater than the square of correlations between a pair of research variables [53]. Details related to the measurement model assessment are reported in Table 2.

### 4.2. Proposed Model Improvement

The structural model was determined. Our results from the SEM demonstrated that the goodness-of-fit statistics for the proposed model comprised an insufficient fit to the data (χ^2^ = 1207.897, *df* = 391, *p* < 0.001, χ^2^/*df* = 3.089, RMSEA = 0.083, CFI = 0.902, IFI = 0.903, TLI = 0.891) [52]. According to the modification indices of the SEM results, considering interrelationships among involvement, knowledge value, visitor satisfaction, desire, and visitor loyalty are desirable to increase the goodness of model fit. In order to obtain a parsimony model within the proposed theoretical framework, we added four paths linking key study constructs (i.e., involvement → knowledge value; involvement → desire; knowledge value → visitor loyalty; and visitor satisfaction → visitor loyalty). As shown in Table 3, these newly integrated linkages are all positive and statistically significant (β _Inv.–KV_ = 0.274, *p* < 0.01; β _Inv.–Des._ = 0.350, *p* < 0.01; β _KV– VL_ = 0.152, *p* < 0.05; β _VS–VL_ = 0.253, *p* < 0.01).

**Figure 1:**
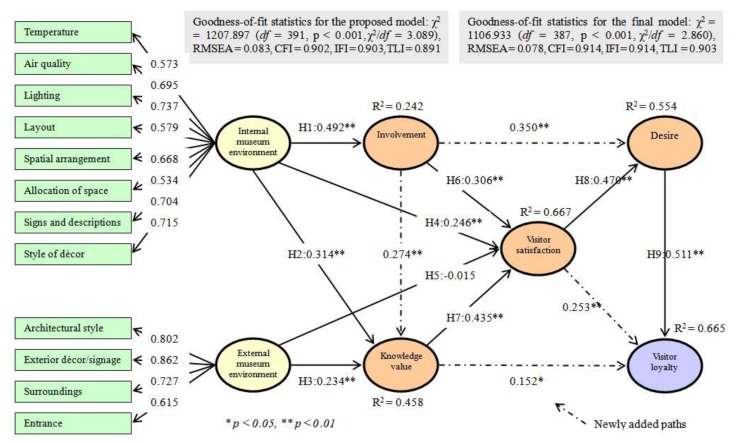
Structural model results.

The chi-square difference test results disclosed that the addition of these paths also significantly improved the model fit (Δχ^2^ (4) = 100.964, *p* < 0.01). The revised model included a satisfactory fit to the data (χ^2^ = 1106.933, *df* = 387, *p* < 0.001, χ^2^/*df* = 2.860, RMSEA = 0.078, CFI = 0.914, IFI = 0.914, TLI = 0.903). Moreover, these added links are theoretically and empirically supported by previous studies [1,4,20,24,39]. In particular, Skadberg and Kimmel [39] indicated that individuals’ involvement experiences increase his/her learning and influences his/her attitudes and behavior. Dirsehan [1], Chen and Chen [54], and Siu et al. [4] identified that these cognitive variables also exerted a significant influence on evaluative, motivational, and conative/loyalty processes in explaining human behavior. Brida et al. [20] asserted that individuals’ satisfactory museum experiences significantly trigger their loyalty. Lastly, the revised model has a significantly greater sufficiency in accounting for the total variance in visitor loyalty (R^2^ = 0.665) than the original model (R^2^ = 0.600). Accordingly, this revised final model was maintained for hypotheses testing and further analysis. This final model is exhibited in Figure 1.

### 4.3. Hypotheses Testing

The proposed impact of the internal museum environment on involvement and knowledge value was tested (Hypothesis (H) 1 and H2). Results showed that both involvement (β = 0.492, *p* < 0.01) and knowledge value (β = 0.314, *p* < 0.01) were a significant and positive function of internal museum environment, which supports Hypotheses 1 and 2. Hypothesis 3 was tested. The relationship between external museum environment and knowledge value was found to be significant (β = 0.234, *p* < 0.01) and such outcome supported Hypothesis 3. The proposed impact of internal and external museum environments on visitor satisfaction was evaluated (H4 and H5). Our findings revealed that while the internal museum environment exerted a significant influence on satisfaction (β = 0.246, *p* < 0.01), external environment (β = −0.015, *p* > 0.05) was not significantly associated with satisfaction. Hence, the results supported Hypothesis 4 but not Hypothesis 5. Hypotheses 6 and 7 were tested. As expected, both involvement (β = 0.306, *p* < 0.01) and knowledge value (β = 0.435, *p* < 0.01) significantly and positively affected visitor satisfaction. Thus, Hypotheses 6 and 7 were supported. The hypothesized links between satisfaction and desire and between desire and visitor loyalty were evaluated. The results indicated that visitor satisfaction exerted a substantial impact on desire (β = 0.470, *p* < 0.01), and visitor loyalty was a noteworthy function of desire (β = 0.511, *p* < 0.01). This finding supported Hypotheses 8 and 9. The variables within the proposed theoretical framework explained 24.2%, 45.8%, 66.7%, and 55.4% of the total variance in involvement, knowledge value, visitor satisfaction, and desire, respectively.

### 4.4. Indirect and Total Impact Assessment

The mediating role of research variables was examined. An investigation of the mediating framework within a theoretical model contributes to a better understanding of the complex associations among research constructs [35]. The outcomes of the indirect and total impact assessment are presented in Table 4. Findings indicated that internal (β = 0.345, *p* < 0.01) and external museum environments (β = 0.102, *p* < 0.01) meaningfully influenced visitor satisfaction indirectly via involvement and knowledge value. That is, involvement and knowledge value served as a key mediating role in the physical environments–satisfaction relationship. Our results also showed that involvement (β = 0.200, *p* < 0.01) and knowledge value (β = 0.204, *p* < 0.05) had a significant indirect influence on desire through visitor satisfaction, and this satisfaction had a significant indirect effect on visitor loyalty through desire (β = 0.240, *p* < 0.01). In other words, both satisfaction and desire acted as significant mediators in the proposed theoretical framework.

Subsequently, we assessed the total impact of the research variables. As presented in Table 4, the results of the structural analysis revealed that desire (β = 0.511, *p* < 0.01) had the utmost impact on visitor loyalty, followed by satisfaction (β = 0.493, *p* < 0.01), internal environment (β = 0.448, *p* < 0.01), involvement (β = 0.430, *p* < 0.01), knowledge value (β = 0.367, *p* < 0.01), and external environment (β = 0.078, *p* > 0.05). Moreover, the findings indicated that the relative importance of involvement (β = 0.549, *p* < 0.01), satisfaction (β = 0.470, *p* < 0.01), and internal environment (β = 0.450, *p* < 0.01) in generating desire was greater than other study variables. Lastly, internal museum environment was identified to be the greatest contributor in increasing visitor satisfaction (β = 0.591, *p* < 0.01).

## 5. Discussion and Implications

Clear comprehension of visitors’ loyalty formation for museums is one of the crucial elements in developing effective marketing/service strategies for visitor retention and revenue increases. The present study successfully integrated such imperative concepts as physical environment, involvement, knowledge value, satisfaction, and desire into one theoretical and conceptual framework of visitor loyalty in the museum industry and explored the complicated interrelationships among these pivotal factors. Our empirical findings enhanced our understanding of personal decision-making and loyalty generation processes through the use of a rigorous and integrative conceptual framework in the domain of museum visitor behavior.

The total impact of internal museum environment on its outcome variables (knowledge value: β = 0.449, *p* < 0.01; visitor satisfaction: β = 0.591, *p* < 0.01; desire: β = 0.450, *p* < 0.01; visitor loyalty: β = 0.448, *p* < 0.01) was pivotal. Our results imply that designating and managing diverse attributes of internal physical environment nurture visitors’ involvement, learning in museums, satisfaction, desire, and loyalty. Given this, a marketing/operational strategy should involve ensuring a quality internal physical environment in museums. As indicated in this study, to fortify museum visitors’ loyalty by taking advantage of visitor involvement, knowledge value, satisfaction, and desire, industry practitioners in museums should put considerable effort and resource investments into enhancing physical-environment conditions. For instance, providing comfortable temperatures, improving the indoor air quality, offering comfortable natural and artificial lighting, improving the layout, making adequate spatial arrangement of the exhibits, improving the décor, signs and descriptions, and providing adequate spaces for programs, gathering areas, catering, and gift shops would not only make a visitor regard a museum as a user-friendly place but also ultimately lead to an increase in visitor loyalty in the museum industry.

The present research empirically demonstrated that while the major driver of knowledge value and visitor satisfaction and their outcome variables is the internal museum environment, the external physical environment is also a significant contributor eliciting visitors’ value perception and evaluation of the overall museum experience. Previous studies about visitor behavior in the extant museum literature have mainly centered on the aspect of internal physical surroundings [8,13]. The impact of the external museum environment has received comparatively less attention from researchers and practitioners. Our results therefore contain a theoretical meaning in that this study is one of several studies that verified the criticality of external museum environments comprising architectural style, exterior décor and signage, surroundings, and entrances in the visitor loyalty generation process. Our findings discovered that, like the role of internal physical surroundings in helping visitors increase knowledge about art/culture/history and reinforcing their satisfaction evaluation, the external museum environment also helps visitors effectively gain knowledge value and satisfactorily evaluate their overall museum experience. From a practical aspect, diverse endeavors are necessary to improve the convenience of entrances/moving paths and the attractiveness of architectural style, exterior décor, landscaping, etc. Based on these efforts, visitors would have valuable and satisfactory experiences at museums.

The insignificant relationship for the link from external museum environment to visitor satisfaction (*p* > 0.05) was attributable to the full mediating role of knowledge value. That is, visitors’ perceived knowledge value perfectly mediated the impact of external museum atmospherics on their satisfaction (β _EME–KW–VS_ = 0.102, *p* < 0.01). This result is consistent with the work of Siu et al. [4] that proposed and verified the mediating nature of knowledge value in explaining visitors’ behavior in museums. Hence, it is inadequate to conclude that the external museum environment/visitor satisfaction association is not imperative. In our theoretical framework, results related to this association should be interpreted with caution. The discovery of the mediating role of knowledge value theoretically contributes to the research gaps in contradictory importance of external museum environment and fulfills insufficiency of empirical studies in the field of museum context.

Little research has investigated the mechanism underlying the effect of visitors’ assessment of physical environments on their satisfaction with museum experiences. In the present study, we uncovered the mediation mechanism including involvement and knowledge value, which offers additional explanation for such effect. This research identified that involvement and knowledge value as consequences of atmospherics acted as intermediaries linking physical environment factors to museum visitor satisfaction. Our results are consistent with previous studies indicating the mediating nature of learning and intense engrossment in museums [1,4,39]. Overall, our intricate theoretical mechanism found that individuals favorably evaluate their museum experiences because the museum provides involvement experiences and creates value for them by improving their knowledge. This further indicates that such involvement and value grow based on diverse internal and external attributes of the physical environment.

In the increasingly competitive environment of the museum industry, practitioners should invent diverse strategies of increasing visitors’ involvement level and knowledge value, which are necessary for satisfaction enhancement. For instance, according to Dirsehan [1], experiential learning can be encouraged by developing scenarios related to museum subjects/exhibits. For museum operators, actively providing more dynamic learning experiences for visitors by using such experiences as a competitive tool would increase visitors’ perceived level of learning/knowledge and help visitors become intensively absorbed in museum subjects/exhibits. In addition, practitioners may get visitors involved by obtaining a short survey for any suggestions and hosting unique events in the halls or throughout social media platforms such as Facebook, Twitter, and Instagram etc. Many people actively use social networking services (SNS) and are passionate to share their opinion or information. By utilizing SNS culture, museum operators can take advantage of unlimited free advertising resources for sharing/promoting their museum and provide more experiences for visitors. Nevertheless, as demonstrated in this paper, the superlative way to increase the level of involvement and value leading to increased visitor satisfaction can be designing and managing physical environments effectively.

In the present research, satisfaction and desire were identified to be important mediators. Recognizing the mediating nature of these variables, Oliver [24] and Perugini and Bagozzi [48] theorized them as complete mediators when explicating individuals’ decision-making processes. Our result was partially compatible with their findings in that involvement was significantly related to desire both directly and indirectly through visitor satisfaction, and visitor loyalty was associated with satisfaction both directly and indirectly through desire. In other words, unlike our original proposition viewing satisfaction and desire as complete mediators, these variables in a museum context act as partial mediators. Researchers should be aware of this incomplete mediating nature of satisfaction and desire in museums when utilizing them for theory development or extension. Our findings also informed museum practitioners that the degree of the satisfaction and desire of their outcome variables and the influence of the direct antecedents are highly dependent on the level of such mediating variables.

The comparison of relative effectiveness among study variables revealed that desire played a prominent and efficient role in determining visitor loyalty (β = 0.511, *p* < 0.01). Prior studies on marketing and consumer behavior have repeatedly stressed the importance of desire [25,26,48,55]. Our result was supported by these extant studies. Existing museum studies have somewhat overlooked this critical concept of desire. Our results highlighted the significance of the motivational process building loyalty and generating growth. Namely, creating one’s desire is a key aspect to his/her increased loyalty in museums. Our findings also identified that satisfaction is the major driving force of desire. Given this, it seems to be necessary for museum practitioners to offer satisfactory experiences to customers in order to boost the level of desire to visit a museum.

## 6. Limitations

The present research findings provided strong support for our theoretical framework and predicted associations among study variables. Nevertheless, as in other studies, this research included a few limitations. Firstly, we tested the conceptual framework using convenience data of the museum customers in seven museums located in one metropolitan city. Therefore, the generalizability of the study findings may be limited. The generalizability should be warranted by adopting a broader sampling range in future research. Second, Siu et al. [4] indicated that visitors’ perceived level of relationship investment by museum operators is vital in explaining visitor behaviors. In the present study, the role of this important factor was not considered. Future research should integrate the concept of relationship investment to improve the sufficiency of our theoretical framework. Lastly, previous research indicated that visit intensification (e.g., a purchase of gifts/souvenirs in museum shops) is an important post-museum experience dimension [1]. Rojas and Camarrero [56] also asserted that satisfied individuals often intensify their experience while visiting a museum by purchasing tangible or memorable materials related to their visit. In the present study, this essential post experience dimension was not incorporated. An opportunity for exploring further relationships linking our study variables to such visit intensification, therefore, exists for future research.

## 7. Conclusions

Our study tries to develop a visitors’ loyalty model for museums by considering the role of internal and external physical surroundings, cognitive processes, evaluative procedures, and motivational processes. A structural analysis was applied to assess the proposed model and examine the hypothesized associations among constructs. The original model was amended by integrating four paths linking key variables within the proposed theoretical framework. The revised final model generated through this modification process included a better fit and explanatory power for visitor loyalty. Internal and external museum atmospherics were identified to be important in the model, and other research variables were found to be direct and indirect driving forces of loyalty. Overall, our conceptual framework involving seven essential constructs in museums and 13 paths relating such variables was sufficiently capable of explaining visitor loyalty formation. In addition, our specific goals, attempted to fill five major gaps, were achieved in the museum context.

## Figures and Tables

**Table 1:** Measurement items and loadings.

Measures	Standardized Loading	Composite Reliability	Cronbach Alphas
IME1: The temperature of this museum was comfortable.	0.591	0.858	0.858
IME2: The air quality of this museum was appropriate.	0.717		
IME3: The natural and artificial lighting of this museum was appropriate.	0.755		
IME4: The layout made it easy for me to move around.	0.581		
IME5: The spatial arrangement of exhibits was appropriate.	0.666		
IME6: The allocation of space for programs, gathering areas, catering, and gift shops were appropriate.	0.524		
IME7: The signs and descriptions were properly located.	0.689		
IME8: The style of décor was high quality.	0.705		
EME1: The architectural style was visually attractive.	0.804	0.841	0.812
EME2: The exterior decoration and signage were visually attractive.	0.860		
EME3: The surrounding of this museum was visually appealing (e.g., landscaping, entrances, surrounding area).	0.726		
EME4: The entrance was conveniently located.	0.614		
Inv.1: I was intensely absorbed in the exhibits.	0.843	0.924	0.922
Inv.2: My attention was focused on the exhibits.	0.925		
Inv.3: I was deeply engrossed in the exhibits.	0.918		
KV1: Visiting this museum enhanced my knowledge about art, culture, and history.	0.902	0.943	0.947
KV2: I acquired new knowledge about art, culture, and heritage through my visit at this museum.	0.933		
KV3: My visits to this museum helped me gain knowledge about art, culture, and heritage.	0.889		
KV4: Visiting this museum increased my knowledge about art, culture, and heritage.	0.867		
VS1: Overall, I am satisfied with my experience at this museum.	0.883	0.931	0.930
VS2: My decision to visit this museum was a wise one.	0.926		
VS3: As a whole, I have really enjoyed myself at this museum.	0.903		
Des.1: I desire to revisit this museum in the near future (False—True).	0.932	0.954	0.954
Des.2: I have a strong desire to revisit this museum in the near future (Very weak—Very strong).	0.930		
Des.3: I want to revisit this museum in the near future (False—True).	0.943		
VL1: I will revisit this museum in the near future.	0.893	0.920	0.934
VL2: I plan to revisit this museum in the near future.	0.929		
VL3: I will make an effort to revisit this museum in the near future.	0.932		
VL4: I would spread positive word-of-mouth about this museum.	0.762		
VL5: I plan on recommending this museum to my family, friends, or others.	0.731		

Note 1. IME = Internal Museum Environment, EME = External Museum Environment, Inv. = Involvement, KV = Knowledge Value, VS = Visitor Satisfaction, Des. = Desire, VL = Visitor Loyalty. Note 2. Measures for all study variables except for desire were assessed with a seven-point Likert scale from “Extremely disagree” (1) to “Extremely agree” (7). Note 3. All standardized loadings were significant (*p* < 0.01).

**Table 2:** Measurement model results (*n* = 305).

	IME	EME	Inv.	KV	VS	Des.	VL	AVE
**IME**	1.000							0.433
**EME**	0.605 ^a^ (0.366) ^b^	1.000						0.573
**Inv.**	0.420 (0.176)	0.409 (0.167)	1.000					0.803
**KV**	0.523 (0.274)	0.523 (0.274)	0.502 (0.252)	1.000				0.807
**VS**	0.562 (0.316)	0.493 (0.243)	0.615 (0.378)	0.694 (0.482)	1.000			0.818
**Des.**	0.375 (0.141)	0.379 (0.144)	0.617 (0.381)	0.525 (0.276)	0.656 (0.430)	1.000		0.874
**VL**	0.450 (0.203)	0.456 (0.208)	0.560 (0.314)	0.651 (0.424)	0.766 (0.587)	0.746 (0.557)	1.000	0.701
**Mean (SD)**	4.897 (0.845)	4.986 (1.015)	4.639 (1.058)	5.062 (1.042)	5.044 (1.086)	4.404 (1.239)	4.755 (1.208)	
Goodness-of-fit statistics for the measurement model: χ^2^ = 1092.539 (*df* = 380, *p* < 0.001, χ^2^/*df* = 2.875), RMSEA = 0.079, CFI = 0.915, IFI = 0.915, TLI = 0.902								

Note. IME = Internal Museum Environment, EME = External Museum Environment, Inv. = Involvement, KV = Knowledge Value, VS = Visitor Satisfaction, Des. = Desire, VL = Visitor Loyalty, SD = Standard Deviation. ^a^ Correlations between constructs are below the diagonal. ^b^ Squared correlations between constructs are within parentheses.

**Table 3:** Structural model results (*n* = 305).

Hypotheses	Links	Coefficients	t-Values
H1	IME → Inv.	0.492	6.304 **
H2	IME → KV	0.314	3.482 **
H3	EME → KV	0.234	2.943 **
H4	IME → VS	0.246	3.045 **
H5	EME → VS	−0.015	−0.233
H6	Inv. → VS	0.306	5.992 **
H7	KV → VS	0.435	7.522 **
H8	VS → Des.	0.470	7.821 **
H9	Des. → VL	0.511	8.921 **
Newly added path	Inv. → KV	0.274	4.749 **
Newly added path	Inv. → Des.	0.350	5.770 **
Newly added path	KV → VL	0.152	2.541 *
Newly added path	VS → VL	0.253	3.456 **
Explained variance for the proposed model: R^2^ (VL) = 0.600 R^2^ (Des.) = 0.517 R^2^ (VS) = 0.672 R^2^ (KV) = 0.429 R^2^ (Inv.) = 0.271	Explained variance for the final model: R^2^ (VL) = 0.665 R^2^ (Des.) = 0.554 R^2^ (VS) = 0.667 R^2^ (KV) = 0.458 R^2^ (Inv.) = 0.242	Goodness-of-fit statistics for the proposed model: χ^2^ = 1207.897 (*df* = 391, *p* < 0.001, χ^2^/*df* = 3.089), RMSEA = 0.083, CFI = 0.902, IFI = 0.903, TLI = 0.891	Goodness-of-fit statistics for the final model: χ^2^ = 1106.933 (*df* = 387, *p* < 0.001, χ^2^/*df* = 2.860), RMSEA = 0.078, CFI = 0.914, IFI = 0.914, TLI = 0.903

Note 1. IME = Internal Museum Environment, EME = External Museum Environment, Inv. = Involvement, KV = Knowledge Value, VS = Visitor Satisfaction, Des. = Desire, VL = Visitor Loyalty. Note 2. The chi-square difference between the final model and the proposed model was significant (Δχ^2^ (4) = 100.964, *p* < 0.01). * *p* < 0.05, ** *p* < 0.01.

**Table 4:** Indirect and total impact assessment.

	IME	EME	Inv.	KV	VS	Des.
Knowledge value	0.135 *	–	–	–	–	–
	(0.449)					
Visitor satisfaction	0.345 **	0.102 **	0.119 **	–	–	–
	(0.591)	(0.087)	(0.425)	(0.435)		
Desire	0.450 **	0.041	0.200 **	0.204 *	–	–
	(0.450)	(0.041)	(0.549)	(0.204)	(0.470)	
Visitor loyalty	0.448 **	0.078	0.430 *	0.214 **	0.240 **	–
	(0.448)	(0.078)	(0.430)	(0.367)	(0.493)	(0.511)

Note 1. IME = Internal Museum Environment, EME = External Museum Environment, Inv. = Involvement, KV = Knowledge Value, VS = Visitor Satisfaction, Des. = Desire, VL = Visitor Loyalty Note 2. The total impact of the study variables are in parentheses. * *p* < 0.05, ** *p* < 0.01.
